# Magistral Compounding with 3D Printing: A Promising Way to Achieve Personalized Medicine

**DOI:** 10.1007/s43441-022-00436-7

**Published:** 2022-08-09

**Authors:** Netta Beer, Susanne Kaae, Natalja Genina, Sofia Kälvemark Sporrong, Teresa Leonardo Alves, Joëlle Hoebert, Marie Louise De Bruin, Ingrid Hegger

**Affiliations:** 1 Social and Clinical Pharmacy Research Group, Department of Pharmacy, Faculty of Health and Medical Sciences, University of Copenhagen, Universitetsparken 2, 2100 Copenhagen Ø, Denmark; 2 Manufacturing and Materials Research Group, Department of Pharmacy, Faculty of Health and Medical Sciences, University of Copenhagen, Copenhagen Ø, Denmark; 3 Social Pharmacy Group, Department of Pharmacy, Faculty of Pharmacy, Uppsala University, Uppsala, Sweden; 4 Centre for Health Protection, National Institute for Public Health and the Environment (RIVM), Bilthoven, The Netherlands; 5 Copenhagen Centre for Regulatory Science, Department of Pharmacy, Faculty of Health and Medical Sciences, University of Copenhagen, Copenhagen Ø, Denmark; 6 Division of Pharmacoepidemiology and Clinical Pharmacology, Utrecht Institute for Pharmaceutical Sciences, Utrecht University, Utrecht, The Netherlands

**Keywords:** Compounding, Community pharmacy, Personalized medicine, 3D printing, Regulation, Interviews

## Abstract

**Background:**

Magistral compounding has always been an integral part of pharmacy practice. The increasing demand worldwide for personalized drug treatments might be accommodated by an increase in magistral compounding. The new, flexible technology of 3D medicine printing could advance this process even further. However, the issue of how 3D medicine printing can be implemented within the existing magistral compounding infrastructure has not been explored.

**Aims:**

To investigate how 3D printing can be integrated into the existing compounding system by taking regulatory, economic, and profession-oriented aspects into account.

**Methods:**

Semi-structured interviews were conducted with relevant Dutch stakeholders representing various health institutions, such as health ministries and boards, professional bodies, and different types of pharmacies. Participants were identified through purposeful sampling. Content analysis was applied to identify the main themes.

**Results:**

A total of 15 Dutch stakeholders were interviewed. It was found that the prevalence of compounding in community pharmacies in the Netherlands has decreased as a result of the practice shifting to specialized compounding pharmacies due to higher costs, lack of space, and the need to fulfill quality requirements. All interviewees considered 3D printing to be a promising compounding technique for community pharmacies, as it offers an automated approach with high digital flexibility and enables adapted formulations, including ‘polypills.’ Regulatory and quality assurance challenges were considered comparable to those of normal magistral products; however, there remain pending regulatory issues regarding quality control, particularly for Active Pharmaceutical Ingredients containing intermediate feedstock materials (e.g., prefilled cartridges) in 3D printing. 3D printing was believed to become cost effective over time.

**Conclusion:**

In the Netherlands, specialized compounding pharmacies have largely taken over compounding activities. 3D printing could be introduced within this system; however, challenges regarding how to regulate prefilled cartridges have yet to be addressed. Compounding using 3D printing in regular community pharmacies could enhance patients’ individualized treatment; however, this activity would require incentives to stimulate the return of compounding to normal pharmacy practice.

## Introduction

Compounding, also known as the preparation of extemporaneous or magistral medicines, has traditionally been an integral task of pharmacies and a particular area of competence for pharmacists. More precisely ‘Magistral compounding/ medicines’ is a term from European legislation, which defines Magistral medicines as any medicinal products prepared in a pharmacy in accordance with a medical prescription for an individual patient [1]. Magistral compounding takes place when available registered medicines produced by the pharmaceutical industry do not meet the needs of the individual patient, for example, in cases of pediatric dosages and dosage forms or the avoidance of allergens [2].

Besides, serving a need for the provision of basic medicines, magistral compounding also seems to lead to a closer relationship between the pharmacist and the patient, as compounding enables the pharmacist to better contribute to the patient’s individualized treatment plan [3, 4]. Furthermore, compounding has been reported to lead to a closer working relationship between pharmacists and physicians [3]. Although most prescribed medicines in the European Union (EU) are currently registered medicinal products manufactured by the industry; in countries such as the U.S.A. and Australia, there seems to be a re-emergence of compounding in pharmacies [2, 3].

Another trend worldwide is the increasing demand for the personalization of medical treatments at the level of the individual patient to improve its efficiency and to reduce side effects. The demand for personalization of medicines is due to the numerous problems in health outcomes today relying on the traditional approach of ‘one-size-fits all’ [5]. One way to achieve personalized medicine is to tailor existing products to the patient, for example, by the use of pharmaco-genomic tests to predict the patient’s susceptibility to them, often referred to as ‘precision medicine.’ Another way is to produce the medicines to fit directly the individual physiological characteristics of the patient, also known as ‘personalized medicine’ [6].

Magistral compounding of medicines in pharmacies could play an important role in accommodating ‘personalized medicine’ in society, as compounding allows for differentiation in dosages between patients as well as dosage forms. There are established definitions and legislative frameworks for compounded medicinal products at the EU level; however, there is no harmonization concerning the details across EU countries, where compounding activity falls under the national competencies of the individual countries. Magistral compounding does not have to comply with the same quality criteria for industrial pharmaceutical production as described in the Good Manufacturing Practices (GMP) guidelines [7]. Compounding nevertheless requires quality assurance, facilities, equipment, expertise, staff, and time, thus making it a costly activity for community pharmacies [1].

To advance ‘personalized medicine’ in society, for example, through compounding, new manufacturing techniques should be considered. One such promising new technology is three-dimensional printing (3D printing) [8–10]. This technology can adapt to precise dosage and drug delivery systems according to individual patient needs, by building complex release systems that take into account, for example, how quickly patients metabolize a drug [11]. It can also be used to create ‘polypills’ that combine multiple active pharmaceutical ingredients (APIs) into a single dosage unit [12, 13] and allows for the creation of tablets of different shapes, sizes, colors, and flavors, which could improve medication adherence [14, 15]. 3D printing is thus a flexible technology in which solid dosage forms are digitally designed by special software [16]. In closed printing equipment, cartridges or other relevant feedstock materials containing the formulated ingredients, including API, are used to print the dosage forms (‘units/tablets’). Dosage forms are built by adding materials either in solid, semisolid, or liquid states layer by layer according to the programmed design [16].

The technology is still under development and currently focuses on hardware (printers), software, and specific pharmaceutical issues, such as finding the appropriate excipients to yield the optimal accuracy and precision, release of APIs, and stability [17]. However, the 3D printing technology has been already tested in the hospital settings to make different dosages and ensure validation of the method [10]. Benefits of 3D printing have been demonstrated as compared to the existing compounding [8, 9].

Soon, 3D printing could offer an automated, sophisticated way to produce medicinal products according to specifications and therefore also facilitate a new era for magistral compounding, to accommodate the need of society to create personalized medicine for patients [18]. However, the question of how 3D printing could be implemented into the existing compounding infrastructure in community pharmacies, taking relevant regulatory, economic, and profession-oriented aspects into account, has not been explored. Therefore, the aim of this study was to investigate how 3D printing can be integrated into the existing compounding systems, to enhance the provision of personalized medicines to patients.

## Materials and Methods

The study took place in the Netherlands. In the Netherlands, approximately 5% of prescription medicines in primary care are pharmacy compounded [19], which is relatively high compared to other European countries. Compounding is provided by approximately 350 Dutch community pharmacies, of which approximately 100 pharmacies do so on a regular basis. In addition, there are approximately 20 specialized compounding facilities (“doorleverende apotheken” in Dutch) that have pharmacy status [20]. They function as manufacturing companies and produce mostly officinal (stock) but also magistral (for an individual patient) preparations [21]. Given the extent of Dutch magistral compounding practices and the global trend toward personalized medicine, the case of the Netherlands was found to be relevant for exploring how the new technology of 3D printing could comply with an existing compounding system.

### Design

We opted for an exploratory, qualitative method, as our aim was to investigate how 3D technology could fit into the existing magistral compounding system and to explore how different aspects could affect its implementation. The study was part of a larger study investigating the future role of 3D medicine printing in Europe [20].

Purposeful sampling was carried out by listing all relevant institutions to be included, i.e., actors in the professional fields of compounding and/or future 3D printing, including health institutions such as health ministries and boards, professional bodies, and different types of pharmacies, and asking those institutions to propose a representative. Additional informants were found through the snowball approach.

Data were then collected using semi-structured interviews with the recruited stakeholders. In most cases, interviewees were approached by e-mail and, after indicating interest, received an information letter describing the study’s aim and a consent form. Recruitment was continued until data saturation was reached, i.e., until no new major (or relevant) aspects were presented by participants [22, 23].

The main components of the semi-structured interview guide were the same for all interviews; however, specific issues were highlighted according to the specific expertise of the interviewee. The specific themes of the interview guide pertaining to this study were as follows:

- Current magistral compounding practices: perceived advantages and disadvantages;
- 3D printing: general perceived advantages and disadvantages; and
- How 3D printers could/should be integrated into the existing magistral compounding system.

Most interviews were conducted face-to-face in English by NB, in most cases alongside a Dutch research partner, either IH, TLA, or JH. NB has a background in global health, whereas IH, TLA, and JH have a background in pharmacy. All interviewers had experience in qualitative research, including interviewing [20].

All interviews were audio recorded and transcribed verbatim. The transcription was then reviewed by IH, TLA, JH, MLDB, SKS, or SK for richness and clarity before being sent back to the respondent. The respondents then approved the transcriptions and, in some cases, responded to issues that required further clarification [20].

The transcriptions were imported into NVivo and coded. Content analysis was applied to describe the findings by structuring and classifying them into themes [23, 24]. The analysis was primarily done by NB, while SKS coded some of the interviews. The coded themes were then compared and discussed by the two authors. The themes that emerged during the analysis were as follows:

- The existing magistral compounding system;
- Special issues related to magistral compounding and quality control;
- Reasons for decreased compounding in community pharmacies;
- General perceptions concerning the use of 3D printing for compounding;
- Adoption of 3D printing in the magistral compounding system;
- Regulatory and quality considerations when implementing 3D printing; and
- Special regulatory considerations for raw materials and intermediate feedstock materials.

Written informed consent was obtained from all participants prior to conducting the interview, and participants’ anonymity was ensured throughout the project according to the standards of General Data Protection Regulation (GDPR). The processing and storage of personal data were approved by the Danish Data Protection Agency which is effectively handled by the Faculty of Health and Medical Sciences at the University of Copenhagen (Ref. no.: 514–0342/19–3000).

## Results

From May to November 2019, 14 semi-structured interviews were conducted with a total of 15 Dutch stakeholders (one interview included two respondents). These stakeholders included representatives from the Ministry of Health (VWS); the Medicines Evaluation Board (CBG); the Royal Dutch Pharmacists Association (KNMP); two representatives from the Health and Youth Care Inspectorate (IGJ); the National Health Care Institute (ZIN); the National Institute for Public Health and the Environment (RIVM); a research institute (TNO); an insurance company (Menzis); the International Pharmaceutical Federation (FIP); a public–private partnership **(**Lygature); a hospital pharmacy; a community pharmacy; and a compounding pharmacy. In total, 10 of the 15 respondents were pharmacists by training.

### The Existing Magistral Compounding System

**Fig. 1:**
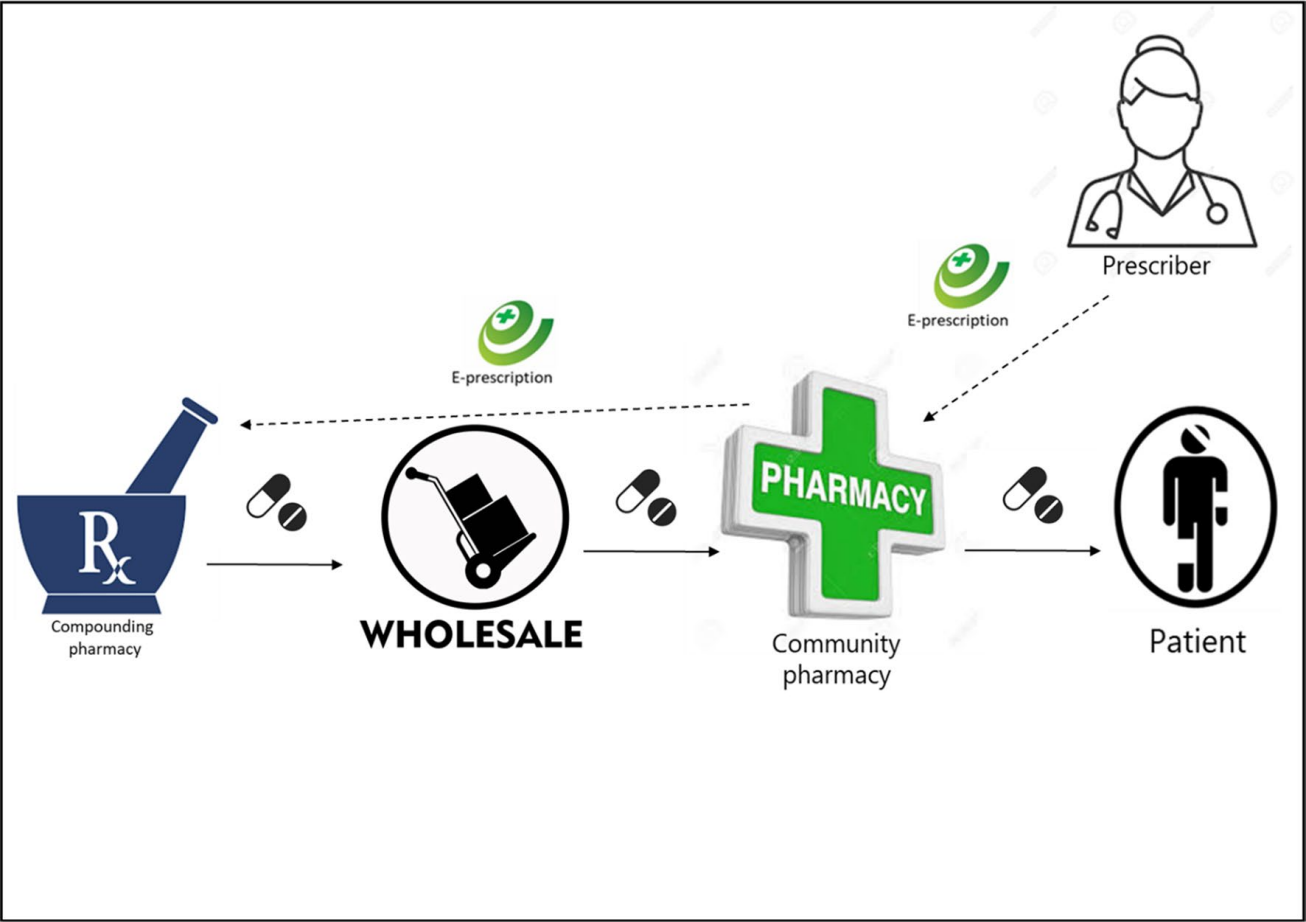
The current distribution of magistral compounded medicines in the Netherlands

In the Netherlands, pharmacies are allowed to compound-registered active APIs as medicinal products. Regulations state that such medicines must be for the pharmacy’s own patients and therefore produced only on a small scale. Respondents mentioned that, in the Netherlands, the production of compounded medicines was declining in hospital and community pharmacies but increasing in specialized compounding pharmacies. Hence, currently, most community pharmacies order compounded medicines from a specialized compounding pharmacy. The order is collected by a wholesaler and delivered to the community pharmacy, which provides it to the patient – see Fig. 1.

Typical reasons for compounding, as stated by the respondents, included the lack of a registered medicinal product available on the market, the need to adjust the dose or excipients including a need for tapering medications, and the need to produce medicines that are otherwise not accessible to the patients, for example, due to high prices.

“I think it’s up to the field to decide [(when to compound]. It could be that ... the formula is not appropriate or the dose is not appropriate. You see nowadays also some hospitals who do it because the price is very high (respon. no. 8).”

Furthermore, the Ministry of Health examined whether compounding facilities could assume a greater role in production when medicine shortages occur; however, this possibility would depend on the availability of APIs and relevant excipients. Most compounded medicines were said not to be solid dosage forms but rather infusions, liquid forms for oral consumption, ointments, and creams. For example, liquid formulations were often compounded for pediatric patients who experienced swallowing difficulties.

### Special Issues Related to Magistral Compounding and Quality Control

There was a general perception among respondents that registered medicinal products were of higher quality than compounded medicines, given the greater control and more standardized procedures involved in the manufacturing process. However, one respondent claimed that this view is not always accurate, especially in the context of medicines for orphan diseases.

Respondents stated that quality control testing for magistral compounding is not as extensive as the testing performed for larger batches of officinal medicines, as it is not possible to perform all the destructive tests needed for quality control. In these cases, the normal testing procedure for solid dosage forms is to weigh the capsules to check for mass uniformity. While there is a legal obligation for compounded medicines to meet the requirements of the European Pharmacopoeia, the extent to which they need to comply with those standards is not explicit. When a batch or a small stock is produced, there is the option of producing surplus tablets so that destructive tests can be performed and so that the surplus tablets can be sent to KNMP’s reference laboratory for further quality control.

On small scale, well, it depends on how small the small scale is. If you do the preparation for one patient and you have to do an analytical control, then you don’t have any product left for the patient. So it is up to the pharmacist to decide on which scale he/she prepares (respon. no 2).

When testing of the final drug product is not possible, respondents mentioned that pharmacists must rely on in-process controls. Respondents stated that the desired quality can be achieved using quality raw materials, good utilities, and trained personnel, as well as by adhering to protocols and validation procedures. Process validation can include content and mass uniformity tests, flow properties, and testing in-between products before reaching the final compounded medicinal product. Respondents stated that there were standard medicinal products routinely produced by pharmacies for which compounding instructions were validated and made available by KNMP to ensure reproducibility.

It was mentioned that the restrictions of current magistral compounding included the challenge of compounding low-dose medicines, given that a minimal volume of API powder is needed for mixing and that capsules contain many excipients. It was also stated that there needs to be a rationale for combining several APIs into one preparation. Pharmacists were believed to have the training and practical knowledge to decide whether combining was desirable and (chemically) possible.

### Reasons for Decreased Compounding in Community Pharmacies

Despite the fact that some respondents claimed that pharmacists would like to continue compounding to provide their patients with the best individual care possible, many pharmacists have stopped compounding. The respondents attributed this reduction in compounding in smaller hospitals and community pharmacies mostly to the high costs involved in setting up the infrastructure and maintaining a compounding area and equipment as well as the time and labor that compounding requires. Further, respondents stated that many pharmacists have struggled to achieve and sustain the quality standards needed for compounding.

Additionally, respondents stated that many community pharmacies simply lack the space to compound, as they struggle for space to stock medicines. Furthermore, the rise of specialized compounding facilities has provided pharmacies with an alternative to acquire compounded products for their patients. Thus, the need for compounding at community pharmacies has decreased over recent decades. One respondent explained that the demand for compounded medicines has also declined due to a change in policy. There is currently a greater emphasis on avoiding compounding unless absolutely necessary and finding registered alternatives.

In those days, the pharmacists said, ‘If you can make it, we made it,’ but nowadays the pharmacists say … ‘‘We are first going to look at the alternative options (respon. no. 7).’

### General Perceptions Concerning the Use of 3D Printing for Compounding

All respondents held that 3D printing could grant benefits in terms of medicine personalization. As in traditional magistral compounding, dosage strength adjustments were especially mentioned as an advantage of 3D printing, either as specialized dosages for different patients or different dosages for the same patient over time. The latter example would cater to the ever-growing number of patients who need to decrease their use of a medication gradually, for example, in discontinuation of corticosteroids or psychiatric medication. Another adjustment mentioned was the customization of excipients in cases of allergies or (lactose) intolerance.

… the biggest advantage is the versatility that you can have. Not one drug fits.

All, but having different quantities is important (respon. no. 10).

The patient populations most frequently cited as the main beneficiaries of personalized medicine and 3D printing were children, elderly individuals, and patients suffering from metabolic disorders as they require a special tailoring of the excipients used. Polypharmacy patients (i.e., patients who regularly take five or more medicines) and psychiatric patients were especially mentioned in the context of ‘polypills.’

Some respondents mentioned using 3D printing technology to encourage medication uptake, for example, by reducing tablet size or creating an orally disintegrating tablet (ODT). One respondent suggested that when treating an acute epilepsy episode, an ODT could be inserted in the buccal space for immediate effect. Opportunities to print tablets with different release profiles, such as sustained and delayed release, were also mentioned.

Most respondents spontaneously mentioned the possibility of creating personalized ‘polypills’ with 3D printers. While fixed dose drug combinations (FDCs) exist in authorized medicines, participants did mention a limitation of such FDCs, i.e., when the dosage of one API is increased, the dosages of other APIs also increase proportionately. With 3D printing, the dosage of each API could be individually adapted. A few participants were also aware of the possibility of compartmentalizing the various APIs within the tablet to avoid interactions, although they questioned whether it was truly necessary.

You could also, very nicely, come up with slow release or, acid resistance and so on. /…/, you can put different APIs in different layers, so that you could avoid stability issues of the combination together, by just putting a layer in between. So, I think there are great possibilities. On the other hand, I don’t know if we need those possibilities (respon. no. 4).

The potential of 3D printing to improve patient adherence to treatment was mentioned. This goal could be achieved by reducing side effects when tailoring medication to the specific patient, by reducing the number of tablets using ‘polypills’ or by customizing appearance and other characteristics, such as taste, to cater to the patient’s personal preferences.

### Adoption of 3D Printing in the Magistral Compounding System

When asked specifically about the benefits 3D printing could bring to magistral compounding, respondents believed that 3D printing could make compounding easier. Given that 3D printing is automated, it could potentially be more accurate and result in fewer human errors, while conventional magistral compounding is a laborious and time-consuming manual process that requires training and experience. It was also suggested that although the printer would be costly initially, it could eventually cut compounding costs, especially by saving time and labor.

I think the costs are high because you have to take a lot of time, and I think that 3D

printing may then be easier, less time consuming, because the machine can do the work (respon. no. 9).

As described, oral solid dosage forms are seldom compounded in pharmacies. However, 3D printing was described as a possible solution to this issue. Hence, one respondent mentioned that if 3D printing was to make it easier to compound solid formulations, it might increase the array of solid products that can be adapted to a specific dose.

The potential role of 3D printing in overcoming drug shortages was noted, but it was also mentioned that this possibility would depend on the availability of the APIs. Another issue that was discussed was the environmental sustainability of 3D printing due to the reduction in transport requirements (if the medications were produced locally) and the associated savings on packaging materials and logistics. One respondent also mentioned that, since 3D printing was a digitalized technique, it could give rise to future opportunities for linking the use of medicinal products with patient data.

Although some respondents argued that 3D printing could save time on compounding, others mentioned that the printers’ speed needed to be improved further. One hospital pharmacist with experience in 3D printing stated that the tasks of preparing the mixture for cartridges and cleaning were time consuming and that the 3D printers that were used currently were thus unable to compete with conventional compounding. An additional disadvantage was pointed out: the need to prevent compounder exposure when dealing with toxic substances, such as teratogenic drugs.

### Regulatory and Quality Considerations When Implementing 3D Printing

Respondents thought that the greatest challenge was the absence of regulatory guidance for 3D printing of personalized medicines. This lack is a matter for authorities to consider when making benefit–risk assessments and evaluating the safety and quality of these compounded medicinal products. Currently, no pharmacopeial monographs for 3D-printed dosage forms exist. The quality control of the final medicinal products will, according to respondents, likely consist of, e.g., tablet weight and dimension checks, similar to conventional compounding at the individual scale. Respondents mentioned the need to validate the printer’s compounding process to ensure that the correct size, weight and, consequently, dose of the dosage form are produced. Furthermore, the stability and other quality aspects of the final medicinal product should be consistent and have the required quality attributes, such as disintegration, dissolution, friability, and bioavailability.

The validation of 3D printing of medicine was described to include quality assurance for the raw materials or intermediate feedstock materials (e.g., prefilled cartridges), including stability and homogeneity; validation of the software and design files; printer validation, including inspection of the printer nozzle and, if heating is involved, temperature validation to preclude overheating; ensuring the correct size or thickness of the layers to be printed; and standardizing cleaning procedures. Respondents were nonetheless unsure about exactly how these processes could be validated. It was stated that the use of validated standard formulations, as currently provided by KNMP, would no longer be feasible, as validation, e.g., of stability, would not be applicable to a personalized formulation.

And that will be different if you have 3D printing, I guess, because if you want to prepare something for specific patients then the composition will differ per patient and then you will not be able to check this anymore... You cannot validate it on a larger scale (respon. no.9).

As other excipients could be used in 3D printing, additional stability testing of printed medicines was stated to be particularly relevant. Additionally, the stability of the APIs could be affected by increased processing temperature, which is needed for 3D printing technology, such as fusion deposition modeling. For other printing techniques, especially the powder bed technique, the mechanical properties (friability) of the dosage form might pose a problem. Additional concerns regarding impurities, side products, and water content were also mentioned. When printing ‘polypills,’ interactions between APIs and the excipients in use would need to be checked. One respondent suggested the creation of a database with compounds that could be combined in 3D-printed tablets.

Finally, there were concerns about reliance on the automated printing process and whether the pharmacist would have control over what was happening in the printer.

…the weak link in that is whether the pharmacist that is using the printer and the databases are equipped enough to ask the proper questions. To make sure whether the device is operating as it should be or is he buying a black box and pushing the button. And that’s my worry… (respon. no. 2)

Safeguards, such as traceable barcodes on prefilled cartridges, were suggested to decrease errors in implementation and increase the safety of printed tablets.

### Special Regulatory Considerations for Raw Materials and Intermediate Feedstock Materials

Respondents explained that the starting materials allowed for compounding are currently either raw materials or registered medicinal products. They claimed that starting with raw materials would be preferable, as purity and quantity are more reliable in this case.

**Fig. 2:**
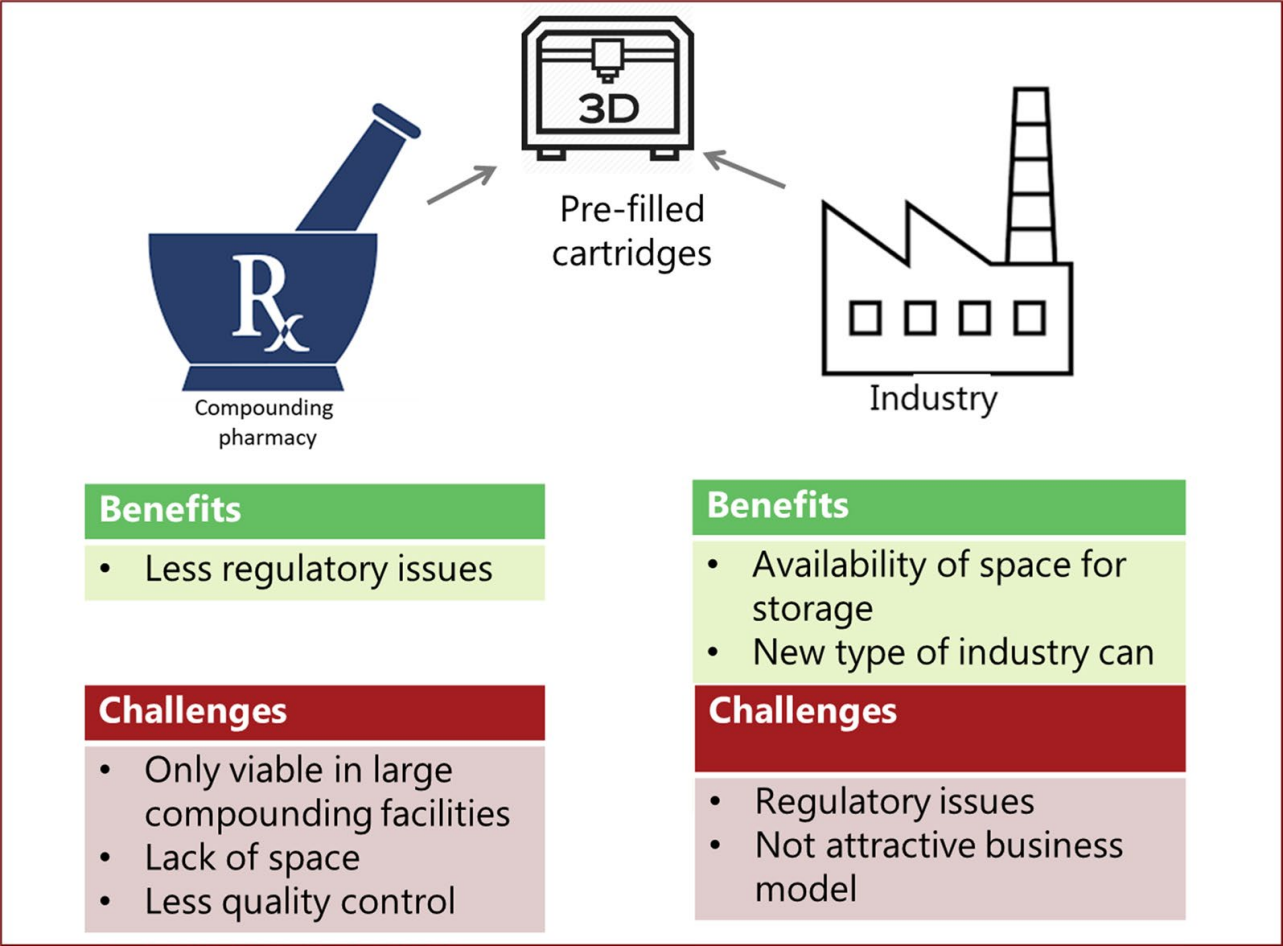
The proposed options concerning where intermediate feedstock materials (e.g., prefilled cartridges) should be prepared and the identified benefits and challenges of each production site

When using 3D printing, cartridges filled with APIs and excipients or other intermediate feedstock materials are inserted into the printers. There were different opinions as to who should prepare these cartridges and the implications of that question on organizational and regulatory aspects. Respondents suggested that cartridges could either be prepared in the pharmacy setting using raw materials or purchased as prefilled cartridges from the industry. The former option of starting with raw materials had no regulatory implications but was perceived as a viable option only in larger compounding facilities. In smaller pharmacies, this option was deemed less feasible due to lack of storage (see Fig. 2).

“If you want to make everything you need to have a lot [of] active ingredients. How do you store it in the pharmacy? (respon. no. 3)”

Benefits supporting industry-made cartridge production were mentioned, such as the possibility of improving the quality control of cartridges and embedding safety controls, such as traceable barcodes, in the cartridges. However, some respondents, particularly those representing health authorities, warned about likely regulatory barriers for industry when trying to obtain marketing authorization for cartridges (see Fig. 2). Current regulations do not have a solution for licensing cartridges, and marketing authorization is currently not required for unfinished products that are not to be supplied directly to patients. These respondents stated that EU legislation refers to “drug substances” and “drug products” but not to “semifinal” products.

…you cannot have an authorization as a medicinal product because a medicinal product should be fit for use by the patient (respon. no. 8).

Nevertheless, respondents stated that intermediate feedstock materials such as prefilled cartridges would need to have a legal status, either as APIs or registered products. Some respondents mentioned that semifinal or intermediate products are only allowed on the market when they do not contain an API. A feasible option mentioned by the health authorities’ representatives was to have the prefilled cartridges produced by GMP-approved compounding facilities. In this case, the pharmacy receiving the cartridges would only be allowed to print tablets for its own patients.

If the 1st big compounding facility delivers it to the 2nd pharmacy, it is allowed, but the 2nd pharmacy has to deliver the final product directly to the patient (respon. no. 2).

However, this solution was also deemed problematic by other respondents due to the lack of quality standards for cartridges. One respondent added that it was unclear whether the issue of marketing authorization for prefilled cartridges was related to the actual legislation or its interpretation but reiterated the need for European harmonization.

## Discussion

In the Netherlands, according to the respondents, compounding is often used when a specific licensed medicinal product is unavailable, for instance, to provide adequate liquid formulations to children or to reduce costs from expensive medicinal products. Compounding in regular community pharmacies in the Netherlands appears to have shifted to specialized compounding pharmacies. This shift was perceived to be a result of high costs, lack of space, and challenges dealing with quality controls. Respondents found the specialized compounding pharmacies to be an appropriate solution to provide individual medicinal products, although this shift entailed less individual care for the patient.

3D printing was seen as a promising compounding technique possessing the same advantages as normal compounding together with additional assets, such as an automated approach with high digital flexibility and the possibility of creating new appropriate personalized formulations. 3D printing was believed to become cost effective over time. Many of the known regulatory/quality challenges for traditionally compounded products were identified; however, pending regulatory issues, especially concerning new quality controls, including controls for intermediate feedstock materials, such as prefilled cartridges, remained.

### 3D-Printed Medicines Integrated into the Existing Magistral System

Our study suggests that a critical issue concerning 3D printing in pharmacies in future will be quality assurance and control. This issue arises mainly because 3D printing will allow a very limited number of personalized dosage forms to be produced for a specific patient and because new tests will have to be introduced. However, several overlaps with traditional compounding were also identified; for example, only a limited number of quality control tests will need to be performed, and in-process controls could as today become extremely important. Quality issues might therefore be relatively easy to resolve through the existing legislation. Furthermore, nondestructive analytical methods, such as different spectroscopic, colorimetric, and image analysis techniques, are already implemented at compounding facilities, especially with the appearance of affordable hand-held instruments [25–27]. This shift can solve the challenge of an insufficient number of analytical tests being performed in the context of 3D printing by ensuring the quality of the produced personalized dosage forms without destroying them (after the tests, the dosage forms can still be delivered to patients). In addition, the long-term stability of personalized dosage forms could be less critical, as those forms are produced for the specific patient and expected to be administered in a relatively short time frame without requiring storage at the pharmacy.

In particular, concerns regarding intermediate feedstock materials such as prefilled cartridges and printers were raised, as they are both new types of intermediaries. It is still unclear where exactly the prefilled cartridges will be produced and how they will be licensed. Further, the validation procedure for printer performance under GMP conditions must still be developed. Finally, the availability of reliable software complying with existing regulatory requirements, such as the Food and Drug Administration’s (FDA) 21CFR Part 11, will be a challenge [28, 29]. Hence, more work still needs to be done to facilitate the introduction of 3D-printed medicines into the existing compounding system.

### 3D-Printed Medicines to Support Optimal Patient Treatment

Respondents in this study found that 3D printing was a relevant compounding technique due to its optimization of patient adherence and other patient outcomes. Studies have shown that patient treatments can be optimized when pharmacists become involved and offer compounding services [4]. This achievement is facilitated not only through the production of individualized medicinal products but also through dialogue between the pharmacist and the patient, during which decisions about the compounded medicinal product are made and the pharmacist explains in depth to the patient how to use the magistral product [3]. If 3D printing is offered, these individual patient dialogues will become even more necessary, as the number of APIs to be combined and the physical appearances of the dosage form, such as color, taste, and shape, all need to be chosen.

For this process to have optimal effects on patients, it could be argued that the task of compounding the medicinal product should be performed by regular individual community pharmacies, as they are the pharmacies who have the contact with patients. This stance is supported by a study by Lobb et al. (2015) [30], who concluded that pharmacists from community pharmacies in which compounding takes place see more advantages in compounding even from the perspective of the patient than do pharmacists from pharmacies where no compounding takes place [30]. Furthermore, pharmacists have reported compounding services to be intellectually stimulating and conducive to a higher sense of responsibility for patient care [4].

The trend in the Netherlands, however, is that compounding has shifted to specialized compounding pharmacies, mainly for economic reasons. Given that 3D printing is believed to become cost effective over time, the question that will arise is whether pharmacy owners will be willing to make the necessary financial investments to return compounding to the community pharmacy level to support patient outcomes. Special incentives to support the process might be needed.

Hence, to induce more personalized medicine to improve outcomes of medical treatments, an increase in compounding is needed. If this should, to some part, rely on 3D printing as a core technique, several initiatives are needed. One initiative is, as explained above, to look into the remaining issues around regulations and the second is creating the most beneficial infrastructure around compounding to harvest all the advantages of the technique.

### Limitations

In order to answer the specific aim of the study, study participants were purposively selected individuals who had experience/knowledge of compounding and/or 3D printing within the pharmacy sector. Therefore, the perspectives of additional relevant actors, such as patients and the pharmaceutical industry, were not investigated. However, this study revealed a variety of relevant aspects of 3D printing as a new compounding technique, which could be explored in a wider societal context in future by conducting supplementary studies with a different type of actors.

Further, the majority of respondents were pharmacists by education, probably because manufacturing of drugs is a special competence by pharmacists. This bias introduced by the purposeful sampling approach might thus have led to certain aspects to the topic being explored more than others, however, also allowed to have the research questions answered in-depth. Further, the interviewed pharmacists represented different types of stakeholder organizations with different needs, thereby reducing the risk of the respondents acting as one united profession.

## Conclusion

In the Netherlands today, when magistral compounding is performed to ensure patients’ access to adequate medical treatment, the compounding often takes place in specialized compounding pharmacies. As both the advantages and limits of traditional compounding also seem, to a large extent, to pertain to the regulatory needs of 3D printing, 3D printing could be introduced into this system. However, one exception for special consideration is the use of intermediate feedstock materials such as prefilled cartridges, which poses additional regulatory challenges. Another exception is the use of printers. The implementation of compounding by 3D printing in regular community pharmacies could support patients’ personalized treatments even further; however, to achieve this outcome, special incentives for the return of magistral compounding to regular pharmacies should be considered.

## Conflict of interest

The authors declare ‘no conflict of interest.’
